# Actively tunable terahertz chain-link metamaterial with bidirectional polarization-dependent characteristic

**DOI:** 10.1038/s41598-019-46436-w

**Published:** 2019-07-09

**Authors:** Pengyu Liu, Zihao Liang, Zhicheng Lin, Zefeng Xu, Ruijia Xu, Dongyuan Yao, Yu-Sheng Lin

**Affiliations:** State Key Laboratory of Optoelectronic Materials and Technologies, School of Electronics and Information Technology, Sun Yat-Sen University, Guangzhou, 510275 China

**Keywords:** Metamaterials, Optoelectronic devices and components

## Abstract

A tunable terahertz (THz) chain-link metamaterial (CLM) is presented, which is composed of a tailored Au layer fabricated on Si substrate. CLM exhibits bidirectional polarization-dependent characteristic by applying a direct-current (dc) bias voltage on device. This CLM device can be heated up the surrounding temperature to tune the corresponding resonance. The tuning range is 0.027 THz from 0.318 THz to 0.291 THz on the bias of 0.60 V to 1.32 V. By reconfiguring the gap between CLM, there are single-resonance with red-shift at TE mode, and multi-resonance with blue-shift and red-shift at TM mode, respectively. These characterizations of CLM are polarization-dependence and bidirectional tunability. These results show the electromagnetic responses of proposed CLM device is suitable for the uses for resonator, filter, switch, and sensor in the THz frequency range.

## Introduction

Terahertz (THz) wave is the transition spectrum from microwave to infrared (IR) wavelength which occupies the spectrum in the frequency range of 0.1 THz to 10 THz. THz metamaterial is an emerging field for electro-optics applications. It is an artificial material can be designed to many kinds of configurations, such as split-ring resonators (SRRs), arrays of subwavelength wires, fishnet structures, etc. Among of these structures, SRRs possess many unique electromagnetic properties which are widely reported to be used in filter, resonator, polarizer, switch, antenna, and sensor^1–5^.

To date, SRRs becomes one of favorite configurations for being metamaterials in a variety of purposes^6^. The traditional SRRs designs are symmetrical or asymmetrical SRRs to realize THz filter, THz polarizer, and THz switch^1^. The configurations are included but not limited to electric SRR (eSRR)^2,7,8^, I-shaped SRR^9^, U-shaped SRR^10^, complementary SRR (cSRR)^11^, V-shaped SRR^12^, and three-dimensional (3D) SRR^13^. To improve the flexibility of THz metamaterial, there are a lot of literatures reported the tuning mechanisms by using semiconductors^14,15^, phase transition materials^16,17^, and superconducting materials^18,19^, stimulated by thermal^20–23^, optical^24,25^, electrical^26^, and magnetic^27^ input signals. These active tuning approaches have been great interest and significance for scientists in real-world applications^20,28–31^.

Recently, reconfigurable metamaterials become feasible in many applications by reconfiguring the dimension of metamaterial unit cell^6^, reshaping the structural elements^14,20^, rotating the unit cell^4,12^ or bending the substrate or lattice^21^, which are other than the uses of thermal control, liquid crystal, phase transition material, and semiconductor diode are highly dependent on the nonlinear properties of the natural materials. These methods suffer from a limited tuning range^31^. The variations in geometrical dimensions of metamaterial structures are often resulted from mechanical movement or metamaterial deformation^2,32^. However, the resonant frequencies of these designs are unaltered, which can only filter or absorb certain electromagnetic spectrum passively. In addition, the reported THz resonators based on SRRs can be only tuned the corresponding resonance with single-directional shift and polarization-independence^4,32^. To develop unique bidirectional polarization-dependent characteristics, it is indispensable to have the abilities of active tunability with large tuning range of resonance.

In this study, we propose an active tuning mechanism to manipulate THz wave by using electrostatic force to perform the reconfigurable metamaterials based on micro-electro-mechanical system (MEMS) technology, which has been widely used in the realization of movable nanostructures and microstructures, providing an ideal platform for the direct reconstruction of metamaterials. The proposed device is a tunable THz chain-link metamaterial (CLM) composed of a tailored Au layer on Si substrate. CLM can be tuned by MEMS technique to realize bidirectional tunability, tunable single-band and multi-band resonances at transverse electric (TE) and transverse magnetic (TM) modes for THz filter and THz polarizer applications. To increase the flexibility of CLM device, the designed CLM can also be actuated by using electrothermal actuation mechanism. It is heating up the near-field temperature to generate the Fano-resonance within CLM. Such change of near-field temperature is accompanied with the refraction index change of surrounding environment. We discuss the relationship of influence for heating temperature and corresponding refraction index of surrounding CLM. Such electromagnetic response of Fano-resonance is artificially created by the induced anisotropic near-field coupling. It can be observed in the resonance tuning by varying environmental temperature of CLM, which creates the possibility to be used in high-efficiency environmental sensor application. This design of MEMS-based CLM opens an avenue to the active tunability of THz waves manipulations with great bidirectional tunability and good polarization-dependence. It can be potentially used in real-world applications such as active sensors, biomedical imaging, flexible electronics, modulators and so on.

## Results

**Figure 1:**
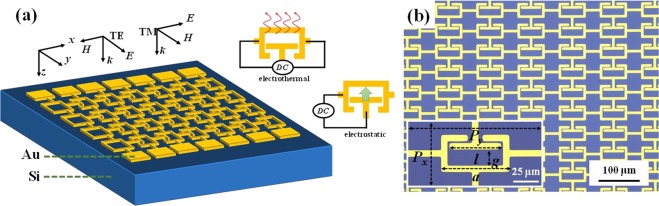
Schematic drawing and optical microscopy image of CLM. (**a**) 3D schematic drawing of CLM device and the coordinates of incident transverse electric (TE) and transverse magnetic (TM) waves, where *E*, *H*, and *k* are the electric field, magnetic field, and wave vector of electromagnetic wave, respectively. (**b**) Optical microscopy image of CLM device and the corresponding denotations of geometrical parameters are indicated in the inserted image of (**b**).

Figure 1(a) shows the schematic drawing of CLM device composed of a tailored Au layer with 300 nm in thickness on Si substrate. The optical microscopy image of CLM device is shown in Fig. 1(b), and the corresponding denotations of geometrical parameters are indicated in the inserted image of Fig. 1(b). The denotations are length of T-shape (*l*), length of SRRs (*a*) and inner distance between T-shape and SRRs (*g*). The period size of CLM is *P*_*x*_ × *P*_*y*_ = 100 μm × 50 μm. The coordinates of incident electromagnetic wave are shown in Fig. 1(a), where *E*, *H*, and *k* are the electric field, magnetic field, and wave vector of electromagnetic wave, respectively.

The proposed CLM devices are adapted using full-field electromagnetic wave simulations performed by commercial simulation software Lumerical, three-dimensional finite difference time-domain (FDTD) solutions. The propagation direction of incident light is set to be perpendicular to the x-y plane in the numerical simulations. Periodic boundary conditions are also adopted in the x and y directions and perfectly matched layer (PML) boundaries conditions are assumed in the z direction. The reflection and transmission of lights are calculated by two monitors set on both sides of device. The electromagnetic resonance of CLM is a function of refraction index of THz wave, according to the theory of Drude-Lorentz model^33^ The corresponding resonance to an external frequency-dependent perturbation can be expressed by

1 $${n}_{EM}=\sqrt{(1-\frac{F\cdot {\omega }_{pe}^{2}}{{\omega }^{2}-{\omega }_{LCe}^{2}})(1-\frac{F\cdot {\omega }_{pm}^{2}}{{\omega }^{2}-{\omega }_{LCm}^{2}})}$$

2 $${\omega }_{LC}=\frac{1}{\sqrt{LC}}=(\frac{{c}_{0}}{S\sqrt{{\varepsilon }_{c}}})\sqrt{\frac{g}{w}}$$

where *ω*_*p*_ is plasma frequency, *ω*_*LC*_ is resonant frequency, *F* is a dimensionless quantity, and *c*_0_ is the light velocity in vacuum. The subscripts *e* and *m* refer to electric and magnetic response. *C* = *ε*_0_*ε*_*c*_*wt/g* and *L* = 2*μ*_0_*S/t* are the respective capacitance and inductance within two designs, where *w* is the metallic width, *g* is the gap width, *t* is the metallic thickness, *S* is the total length of CLM and relative to the *a* parameter, *ε*_0_ is the free space permittivity, and *ε*_*c*_ is the relative permittivity of the materials within CLM gap. Therefore, the resonance of models can be actively tuned by changing the gap width.

**Figure 2:**
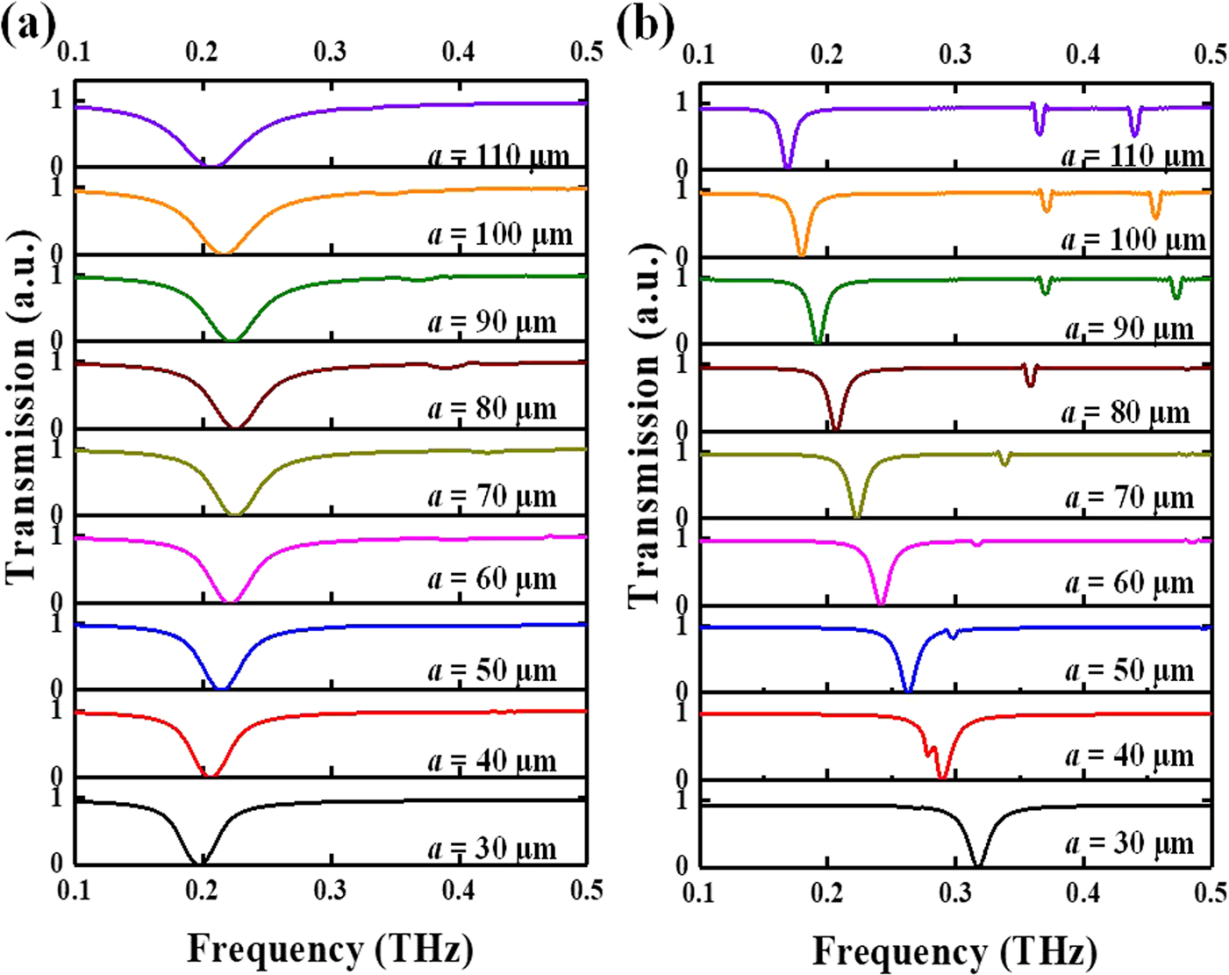
Simulated transmission spectra of CLM with different *a* value. Transmission spectra of CLM with different *a* value at (**a**) TE mode and (**b**) TM mode.

The transmission spectra of model with different *a* value at TE and TM modes are shown in Fig. 2(a,b), respectively. While *l* value is varied along with *a* value simultaneously. The *g* value is kept as constant as 20 μm. At the initial state, resonances of model with *a* = 30 μm are 0.19 THz and 0.32 THz at TE mode and TM mode, respectively. By increasing *a* value from 30 μm to 110 μm, the resonance is blue-shift 0.04 THz first from *a* = 30 μm to *a* = 80 μm, and then red-shift 0.03 THz from *a* = 80 μm to *a* = 110 μm. It is clearly observed that CLM exhibits a bidirectional characterization at TE mode. At TM mode, there is single resonance linearly shifted 0.15 THz from 0.32 THz to 0.17 THz by changing *a* value from 30 μm to 110 μm.

**Figure 3:**
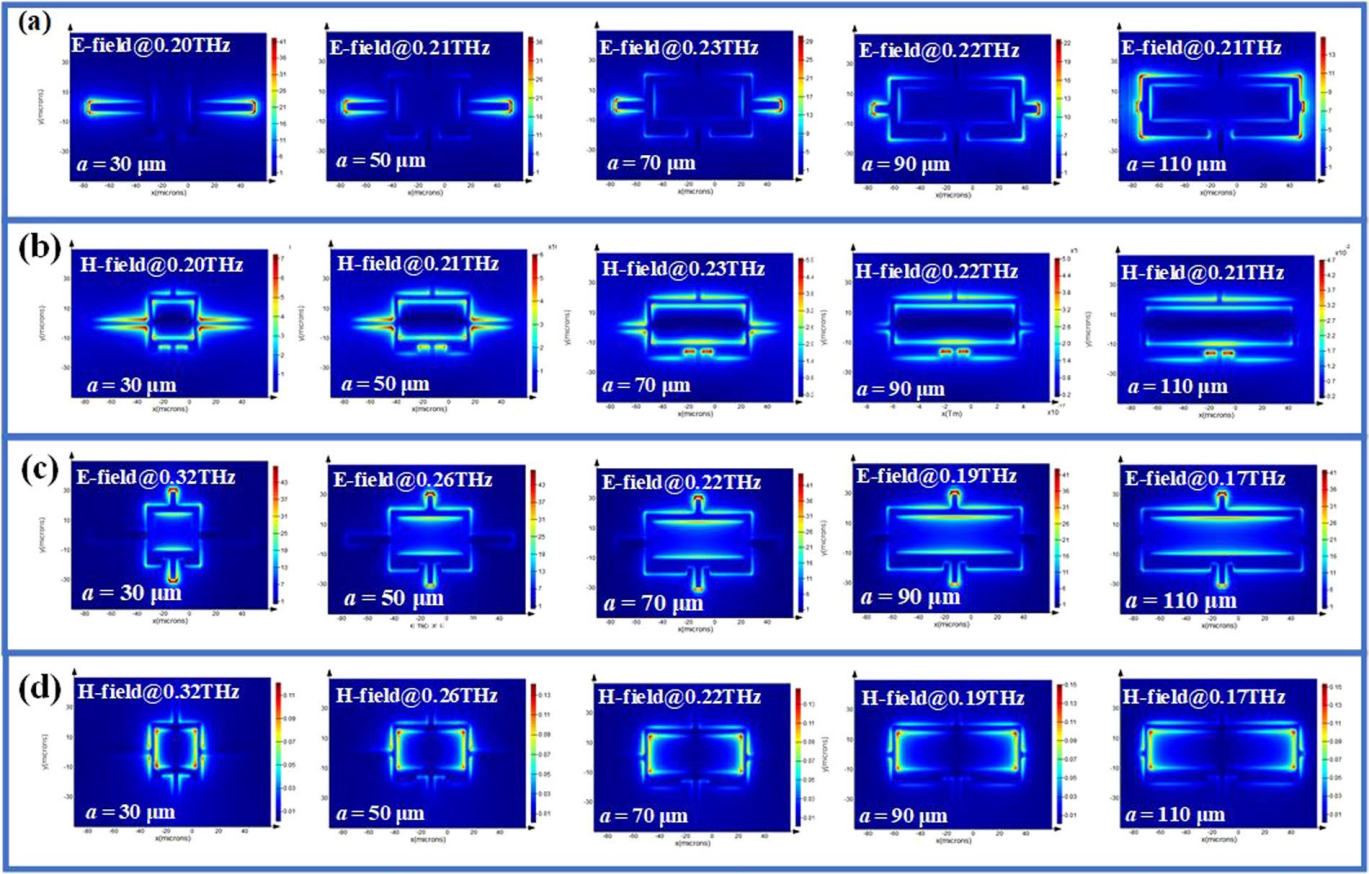
Electromagnetic field distributions of CLM with different *a* value. Electromagnetic field distributed images of CLM with different *a* value at (**a**) and (**b**) TE mode; (**c**) and (**d**) TM mode, where (**a**) and (**c**) are E-field distributions, while (**b**) and (**d**) are H-field distributions. (Symbol @ indicates the corresponding monitored frequency).

To better understand the physic mechanism of electromagnetic response, the corresponding electric (E) field and magnetic (H) field distributions of CLM are monitored, which are indicated in Fig. 3 for TE mode and TM mode, respectively. At TE mode, the E-field energy is distributed on the left and right conductor bars of chain-link microstructures, while the H-field energy is distributed around the top and bottom conductor bars and split of chain-link microstructures. The most of H-field energy is focused within CLM and T-shape metamaterials. At TM mode, the incident THz wave is rotated 90° compared to that at TE mode. Most of E-field energies are distributed around the top and bottom two conductor bars of chain link microstructures, while the H-field is distributed around the inner side of left and right conductor bars of SRR.

**Figure 4:**
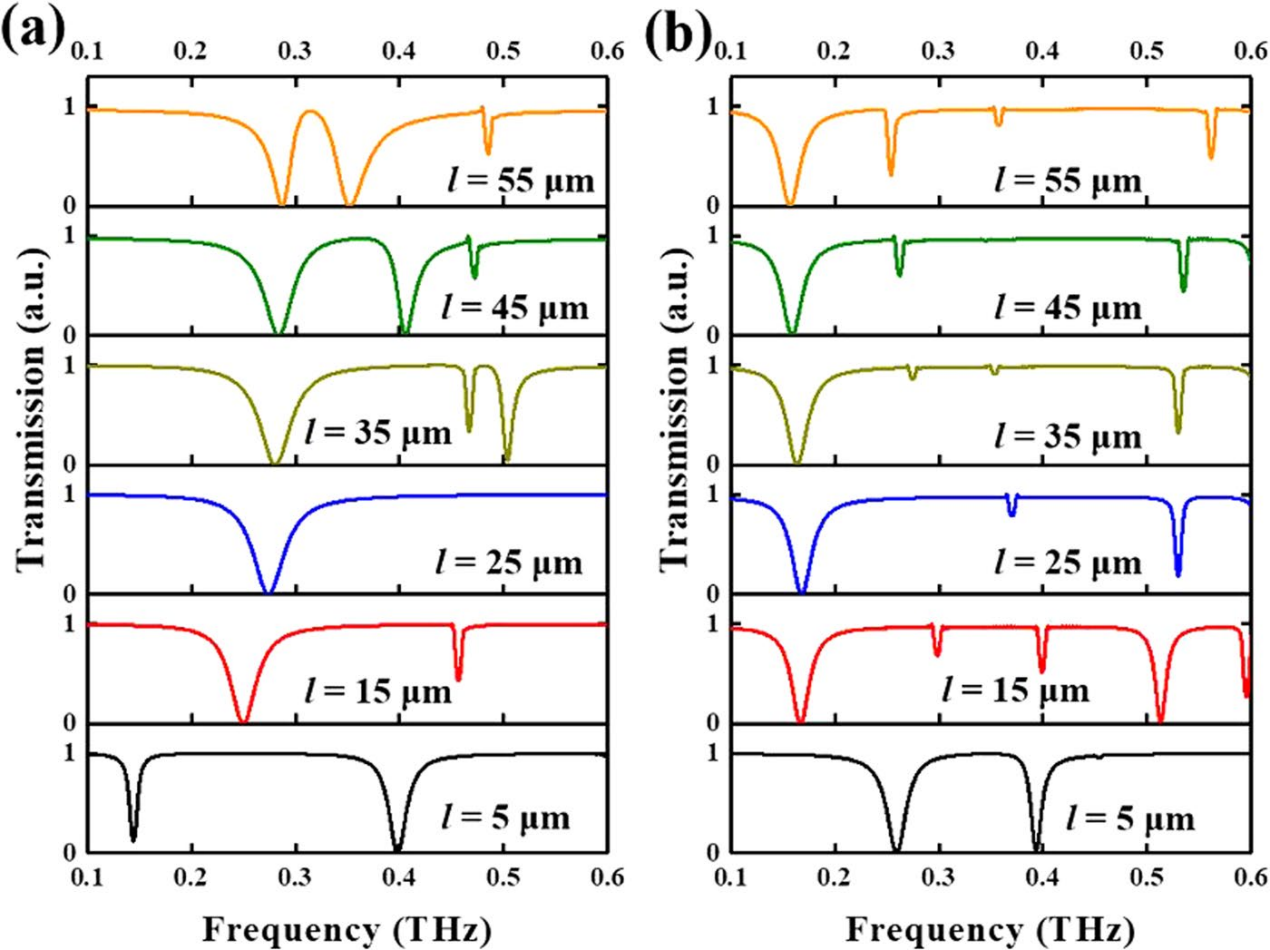
Simulated transmission spectra of CLM with different *l* value. Transmission spectra of CLM with different *l* value at (**a**) TE mode and (**b**) TM mode.

Figure 4 shows the transmission spectra of CLM with different *l* value and other parameters are kept as constant as *a* = 65 μm and *g* = 20 μm at TE and TM modes as shown in Fig. 4(a,b), respectively. At the initial state, resonances of CLM with *l* = 5 μm are 0.14 THz (first resonance) and 0.39 THz (second resonance) at TE mode, respectively. By increasing *l* value from 5 μm to 15 μm, the first resonance is blue-shift 0.11 THz (at 0.25 THz) and second resonance is blue-shift 0.07 THz (at 0.46 THz). When *l* is increasing from 15 μm to 35 μm, a part of the E-field distribution in the split of chain-link microstructures is seperated to the inner contour of SRR. It means there is a transfer for the E-firld energy equally distributed in the split of chain-link microstructures and the inner contour of SRR under the conditions from *l* = 15 μm to *l* = 35 μm. Therefore, it shows the first resonance of CLM with *l* = 25 μm is shift to 0.27 THz, and the second resonance is vanished. By increasing *l* value to 35 μm, the first resonance is shift to 0.28 THz, while there will generate two resonances at 0.47 THz and 0.51 THz. Continuously increasing *l* value to 55 μm, there are three resonances at 0.28 THz, 0.35 THz, and 0.49 THz, respectively. The electromagnetic response of CLM by varying *l* value exhibits the switching function between sing-band, dual-band, and triple-band resonances.

At TM mode, the resonances are 0.26 THz (first resonance) and 0.39 THz (second resonance) at initial state (*l* = 5 μm). By increasing *l* value from 5 μm to 15 μm, the first resonance is red-shift to 0.16 THz and second resonance is blue-shift to 0.52 THz. The resonances are divided into five, which are 0.16 THz, 0.30 THz, 0.40 THz, 0.52 THz, and 0.59 THz, respectively. The dominated resonances are 0.16 THz and 0.52 THz. Continuously increasing *l* value to 55 μm, three dominated resonances are 0.15 THz, 0.26 THz, and 0.56 THz.

**Figure 5:**
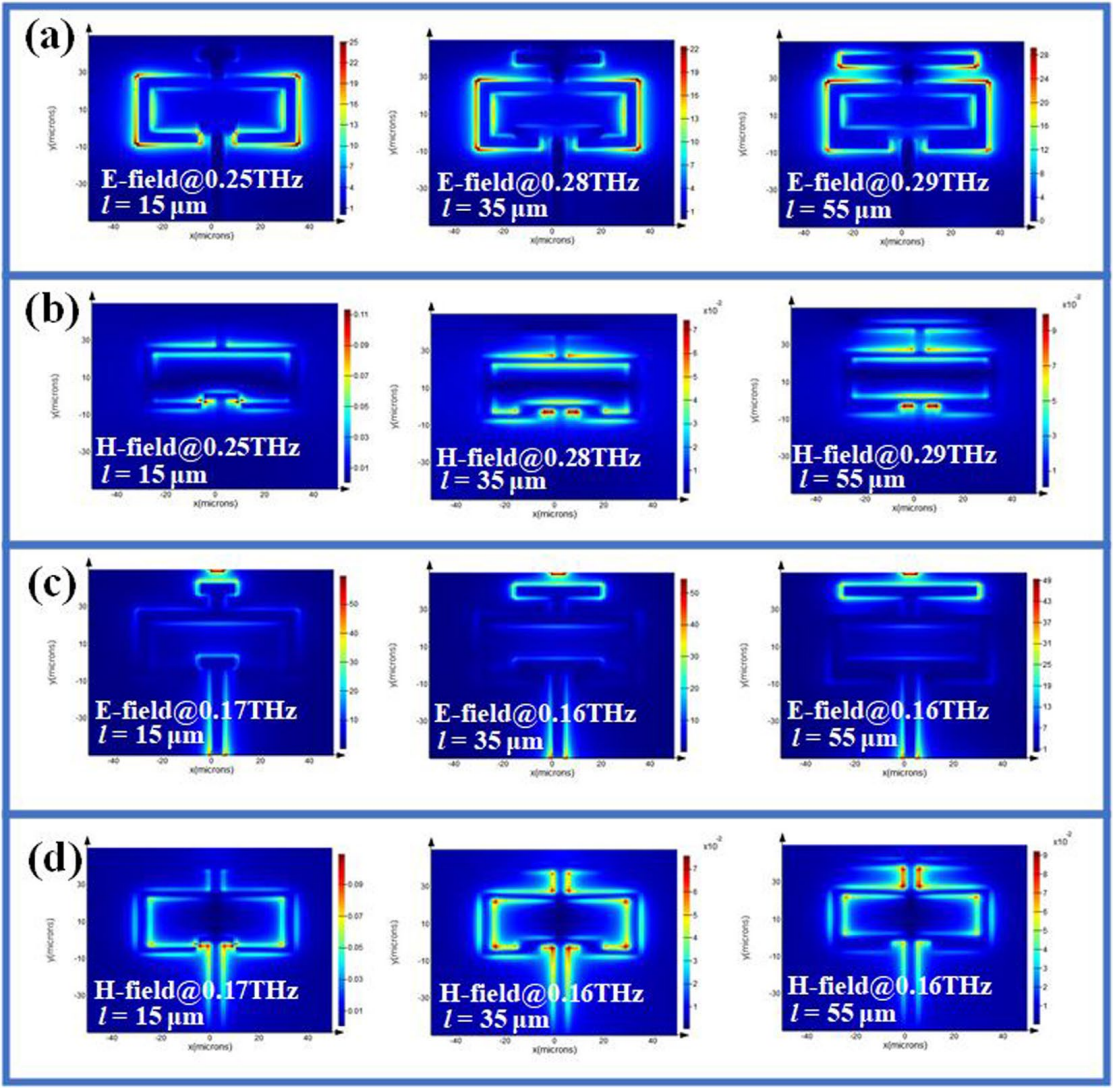
Electromagnetic field distributions of CLM with different *l* value. Electromagnetic field distributed images of CLM with different *l* value at (**a**) and (**b**) TE mode, (**c**) and (**d**) TM mode, where (**a**) and (**c**) are E-field distributions, while (**b**) and (**d**) are H-field distributions. (Symbol @ indicates the corresponding monitored frequency.)

The variations are two, three, four and five resonances at TM mode could be explained by corresponding electromagnetic field distributions indicated in Fig. 5. At TE mode, the E-field energy is distributed on the contour of SRR with two current loops resulted in two resonances, while the H-field energy is distributed around the top and bottom conductor bars and split of chain-link microstructures under the condition of *l* = 15 μm. The most of H-field energy is focused within CLM and T-shape metamaterials.

For the case of *l* = 55 μm, the length of T-shape metamaterial is long enough to make the electromagnetic energy coupling with CLM to form three current loops. At TM mode, the E-field energy is focused on the apexes of CLM and T-shape metamaterials, while the H-filed energy is distributed along the contour of CLM and T-shape metamaterials for the case of *l* = 15 μm. This phenomenon of electromagnetic energy distribution is obvious for the case of *l = *35 μm and *l* = 55 μm. Regarding to above mentioned, the electromagnetic response of CLM exhibits multi-band resonances and polarization-dependence.

**Figure 6:**
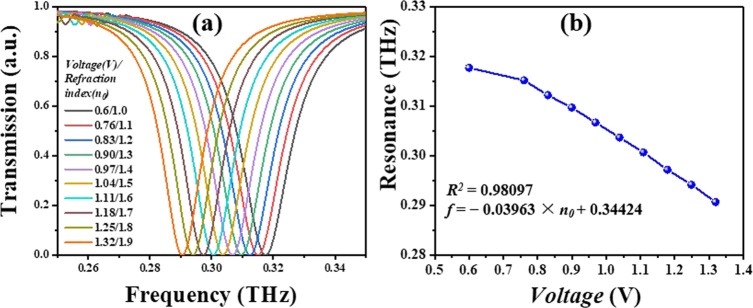
Measured transmission spectra of CLM at different dc bias voltage. (**a**) Transmission spectra of CLM with *a* = 30 μm applied different dc bias voltage (*V*) and calculated corresponding refraction index (*n*_0_) at TM mode. (**b**) is the corresponding relationship of resonance and bias (*V*).

By applying a dc bias voltage on CLM, the surrounding temperature can be heating up. Figure 6 shows the experimental results of CLM applied different bias voltage at TM mode. The resonances of CLM are red-shift with a tuning range of 0.027 THz from 0.318 THz to 0.291 THz. The driving voltage is 0.60 V to 1.32 V. The parameters of CLM device are kept as constant as *a* = 30 μm, *l* = 20 μm, *g* = 20 μm at TM mode applied different dc bias voltage. The resonance is quite linear with a correlation coefficient of 0.98097. These results provide that the proposed CLM device can be used in tunable sensor with single-band resonance characteristic.

**Figure 7:**
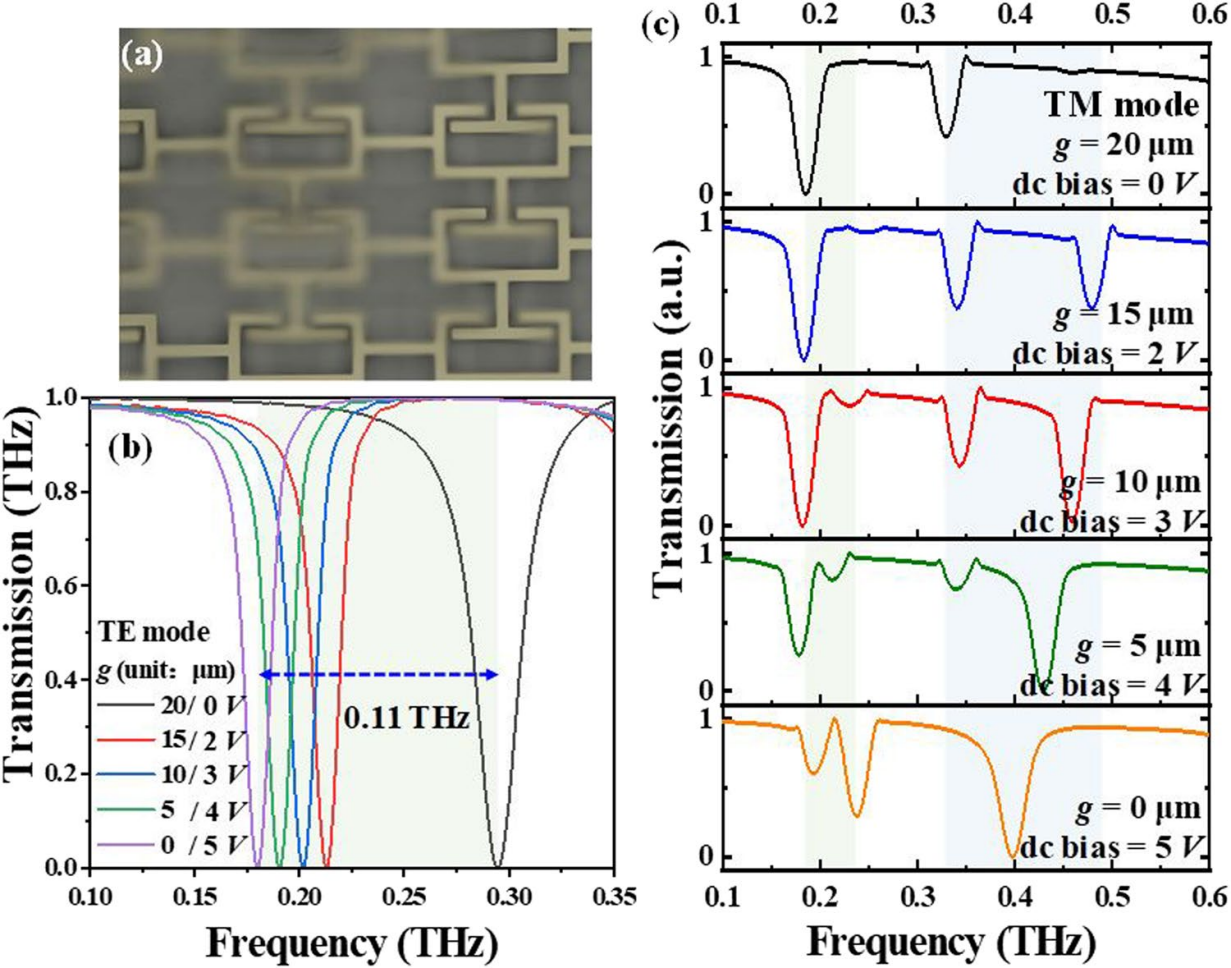
Experimental results of proposed CLM by changing different *g* value. (**a**) Optical microscopy image of released CLM. Transmission spectra of CLM with different *g* value at (**b**) TE mode and (**c**) TM mode.

To further investigate the active tunabilties of CLM device, the gaps between CLMs and T-shape metamaterials are tuned by using electrostatic force to shorten the gaps from 20 μm to 0 μm. Figure 7(a) shows the optical microscopy image of CLM device after released CLMs. The transmission spectra of CLM device with different *g* value by applying different dc bais volatge at TE and TM modes are shown in Fig. 7(b,c), respectively. The geometrical parameters are kept as constant as *a* = 65 μm and *l* = 55 μm. At TE mode, the tuning range of resonance is red-shift 0.11 THz from 0.29 THz to 0.18 THz for *g* = 20 μm to *g* = 0 μm as shown in Fig. 7(b). It exhibits tunable single-band resonance. At TM mode, the initial reso-nances are 0.18 THz (first resonance) and 0.32 THz (second resonance) under the condition of *g* = 20 μm. By tuning *g* value to 10 μm, the first resonance is kept as constant and second resonance is blue-shift to 0.34 THz, while there is generated third resonance around 0.46 THz. By shortening *g* value to 0 μm, the first resonance becomes two resonances, which are 0.19 THz and 0.23 THz while the second and third resonances will merge together at 0.40 THz as shown in Fig. 7(c). Thus, CLM exhibits multi-band with bidirectional polarization-dependent characteristic.

We further demonstrate another active tunabilities by using electrothermal actuation mechanism to facilitate CLM flexibility and applicability. By applying the voltage on both sides of the electrode as indicated in Fig. 1(a), the surrounding temperature will be increased and expressed by

3 $$T-{T}_{0}=P\times {R}_{s}=\frac{{V}^{2}}{{R}_{t}}\times {R}_{s}=\frac{{V}^{2}}{{R}_{0}[1+\alpha (T-{T}_{0})]}\times {R}_{s}$$

where *T*_0_ is the initial room-temperature, *R*_0_ is the resistance at initial room-temperature, *R*_*s*_ is the thermal resistance from substrate to ambient environment, *R*_*t*_ is the resistance at heat-ing temperature, *α* is the linear temperature coefficient of resistance. Owing to the variation of heating temperature can make the surrounding refraction index (*n*_0_) changed, it can be obtained by

4 $$\frac{1}{{n}_{0}}\frac{d{n}_{0}}{dT}=-\,\frac{({n}_{0}^{2}-1){E}_{g}}{({n}_{0}^{2}){E}_{g}^{2}-{(h\gamma )}^{2}}-\frac{3\beta {n}_{0}^{2}-1}{2{n}_{0}^{2}}$$

where *n*_0_ is the surrounding refraction index, *γ* is the frequency of the illumination light, *h* is Planck’s constant, *β* is the thermal expansion coefficient for the material, and *E*_*g*_ is the bandgap energy at any temperature in degrees Kelvin. Furthermore, the ambient refraction index is a key role parameter to determine the Fano-resonance of CLM referred to Eq. (1).

## Discussion

An actively tunable CLM is presented, which is composed of CLMs and T-shape metamaterials on Si substrate. By applying a dc bias voltage on CLM without released CLMs, the tuning range is 0.027 THz with a correlation coefficient of 0.98097. For CLM with released CLMs, the dc bias voltage is applied to change the gap between CLMs and T-shape metamaterials that could be modified and then reconfigured CLM microstructures. The electromagnetic responses exhibit bidirectional polarization-dependence. It can be not only used for THz filter and polarizer applications, but also for single-band to multi-band THz switches. CLM can be actuated by using electrothermal actuation mechanism to heat up the near-field temperature and then make the Fano-resonance of CLM shifting. Such electromagnetic response of Fano-resonance is artificially created by the induced anisotropic near-field coupling. It can be observed in the resonance tuning by applying varying voltage on CLM, which creates the possibility to be used in high-efficiency environmental sensor application.

## Methods

### Numerical simulation

The electromagnetic characteristics of CLM is performed by using a commercial software package (Lumerical FDTD Solutions, ver. 8.19) to study the optical properties of devices. In device simulation, the unit cell structure is used by applying periodic boundary conditions along the x- and y-directions, and the perfectly matched layer (PML) boundary condition along the z-direction. The propagation direction of incident THz wave is perpendicular to the x-y plane in the numerical simulations. The mesh precision is 1 μm with the minimum clearance size of 1 μm. The transmission THz wave is calculated by setting monitor on the bottom side of device.

### Sample fabrication

The proposed structurally CLM device was fabricated using maskless photolithography and lift-off techniques. We proceeded with the deposition of a 10/200 nm thick Cr/Au layer on an unintentional doping Si substrate using a Quprum Q150T ES sputtering system. The deposition conditions were direct-current (dc) Ar plasma at 250 mTorr, gas purity of 99.995%, and discharge current of 50 mA. The sputtering time was controlled to obtain the target thickness. Prior to the sputtering process, the lift-off resistance was spin coated, which was polymethylmethacrylate (PMMA) 950 A2 spun on the device at 4000 rpm for 60 s and baked at 180 °C for 2 min. The metamaterial pattern was defined on the resist layer using a maskless photography technique (WAVETEST Instruments uPG501 System). After development of the resist in 3:1 isopropyl alcohol (IPA)/deionized water (DI) solution for 5 min, the CLM pattern was performed by removing the resist.

### Characterization

With the tunable CLM device, we experimentally explore the electrothermal and electrostatic effects to resonant frequency of CLM device. By increasing the dc bias voltage, the corresponding resonant frequency could be tuned. Our sample was mounted on a home-made printed-circuit board (PCB) with a transparent window. There are two conductive wires soldering on PCB and connected to a dc power supply. The sample on the PCB was placed in the chamber of THz measurement system under a nitrogen environment to eliminate water vapor. The THz transmission spectra characterization was measured using Teraview 3000 time-domain spectroscopy (THz-TDS) system. The transmission spectra were normalized with respect to transmission of pure Si substrate of the same thickness as the sample.

## Data Availability

The authors declare that the data supporting the findings of this study are available within the paper and its Supplementary Information files.
